# Whole-genome sequence of six *Rhizobium laguerreae* strains

**DOI:** 10.1128/mra.00279-24

**Published:** 2024-05-20

**Authors:** Evgenii A. Kirichek, José D. Flores-Félix, Encarna Velázquez, Anna V. Tsyganova, Viktor E. Tsyganov

**Affiliations:** 1 All-Russia Research Institute for Agricultural Microbiology (ARRIAM), Laboratory of Molecular and Cellular Biology, Saint Petersburg, Russia; 2 Departamento de Microbiología y Genética, Universidad de Salamanca, Salamanca, Spain; 3 Instituto de Investigación en Agrobiotecnología (CIALE), Salamanca, Spain; Indiana University, Bloomington, Bloomington, Indiana, USA

**Keywords:** *Rhizobium laguerreae*, genome sequencing, nitrogen fixation

## Abstract

*Rhizobium laguerreae* is regarded as a promising candidate for biofertilization of legume plants worldwide through its high efficiency in symbiosis. In this paper, we report high-quality sequences of six *R. laguerreae* strains with total genome completeness from 93.5% to 97.5%.

## ANNOUNCEMENT

Molecular genetic mechanisms of pea (*Pisum sativum* L.) symbiotic nodule formation are being actively studied (1). Recently, *Rhizobium laguerreae* AMPS strains were isolated from nodules of peas in Aldea de San Miguel (Valladolid) soil in Spain (2). The strains were isolated on plates containing YMA medium (Merck, Spain) and incubated during a week at 28°C according to Vincent (3). The strains were identified via MALDI-TOF MS and analysis of the 16S rRNA, *recA,* and *atpD* genes (4). During laboratory experiments, the high efficiency in symbiosis with peas was shown for these strains (4). However, the genetic determinants underlying such high efficiency have not been discovered yet.

Strains were preserved in tryptone-yeast extract (TY) medium with 40% glycerol at −80°C. For DNA isolation, each strain was revived on a solid TY medium and incubated for 72 h at 28°C. DNA was isolated using the Monarch Genomic DNA Purification Kit T3010L (New England Biolabs, USA) according to the manufacturer’s recommendations without size selection.

Oxford Nanopore Technologies (ONT) sequencing was carried out in the ARRIAM. 1D native barcoding genomic DNA protocol (SQK-LSK109 with EXP-NBD104 and EXP-NBD114) (Oxford Nanopore, United Kingdom) was used to prepare the library according to the manufacturer’s instructions skipping the DNA-shearing step. Sequencing was performed on MinION sequencing device (Oxford Nanopore, United Kingdom) with flow cell R.9.4.1. Base calling, demultiplexing, and barcodes trimming were performed using Guppy base caller v.5.0.1. Quality control of the raw reads was provided by NanoPlot (5) v.1.41.0 (Table 1).

**TABLE 1:** Characteristics of *Rhizobium laguerreae* genome sequences

Strain	AMPS04	AMPS05	AMPS17	AMPS22	AMPS23	AMPS34
Read N_50_ (bp)	7,668	9,494	8,538	16,124	6,804	15,205
# raw reads	69,285	35,261	51,539	61,292	87,548	52,250
Coverage	49×	29×	40×	65×	55×	60×
# contigs	5	8	5	6	6	6
Total length (bp)	7,008,475	7,522,422	6,854,799	7,335,478	7,260,096	7,371,118
Assembly N_50_ (bp)	4,933,869	4,944,891	4,861,011	4,855,452	4,883,801	4,869,780
GC content (%)	60.79	60.75	60.86	60.87	60.71	60.75
# genes	6,847	7,314	6,758	7,112	7,131	7,151
# RNAs	60	61	60	60	61	61
Genome completeness (%)	97	94.1	96.2	95.8	97.5	93.5
Accession #	GCA_036964765.1	GCA_036964845.1	GCA_036964825.1	GCA_036964745.1	GCA_036964785.1	GCA_036967165.1

The genomes were *de novo* assembled using Flye (6) v.2.9.1 with (--nano-raw) option. Further assembly correction was performed using the medaka (7) v.1.7.2. Genome assembly evaluation tool QUAST (8) v.5.2.0 was used to estimate assembly statistics (Table 1). The genome completeness was established using BUSCO (9, 10) v.5.5.0 with the following options: (--mode 'geno' --lineage_dataset 'rhizobium-agrobacterium_group_odb10') (Table 1). Default parameters were used for all listed software unless otherwise noted.

All sequences were deposited at GenBank and annotated using the NCBI Prokaryotic Genome Annotation Pipeline (11) v.6.6 (Table 1).

Obtained assemblies were compared with all represented strains from *R. leguminosarum* complex according to Young et al. (12) using CJ Bioscience’s Orthologous Average Nucleotide Identity Tool (13) v.0.93.1. Thus, AMPS17, AMPS04, and AMPS23 strains belong to the gsR group, AMPS05 and AMPS34 strains belong to the gsO group, and AMPS22 strain belongs to the gsN group.

## Data Availability

All assemblies and sequence data have been deposited in the GenBank under the BioProject accession number PRJNA1079530. The demultiplexed fastq ﬁles, with barcodes removed, from the MinION runs have been deposited in the Sequence Read Archive (SRA) under numbers SRR28063972 (AMPS04), SRR28063971 (AMPS05), SRR28063970 (AMPS17), SRR28063969 (AMPS22), SRR28063968 (AMPS23), and SRR28063967 (AMPS34). This announcement describes the ﬁrst version of the genome assembly.
